# Can Ketones Help Rescue Brain Fuel Supply in Later Life? Implications for Cognitive Health during Aging and the Treatment of Alzheimer’s Disease

**DOI:** 10.3389/fnmol.2016.00053

**Published:** 2016-07-08

**Authors:** Stephen C. Cunnane, Alexandre Courchesne-Loyer, Camille Vandenberghe, Valérie St-Pierre, Mélanie Fortier, Marie Hennebelle, Etienne Croteau, Christian Bocti, Tamas Fulop, Christian-Alexandre Castellano

**Affiliations:** ^1^Research Center on Aging, SherbrookeQC, Canada; ^2^Department of Medicine, Université de Sherbrooke, SherbrookeQC, Canada; ^3^Department of Pharmacology and Physiology, Université de Sherbrooke, SherbrookeQC, Canada

**Keywords:** ketone, Alzheimer’s disease, acetoacetate, beta-hydroxybutyrate, glucose, mild cognitive impairment, aging, medium chain fatty acid

## Abstract

We propose that brain energy deficit is an important pre-symptomatic feature of Alzheimer’s disease (AD) that requires closer attention in the development of AD therapeutics. Our rationale is fourfold: (i) Glucose uptake is lower in the frontal cortex of people >65 years-old despite cognitive scores that are normal for age. (ii) The regional deficit in brain glucose uptake is present in adults <40 years-old who have genetic or lifestyle risk factors for AD but in whom cognitive decline has not yet started. Examples include young adult carriers of presenilin-1 or apolipoprotein E4, and young adults with mild insulin resistance or with a maternal family history of AD. (iii) Regional brain glucose uptake is impaired in AD and mild cognitive impairment (MCI), but brain uptake of ketones (beta-hydroxybutyrate and acetoacetate), remains the same in AD and MCI as in cognitively healthy age-matched controls. These observations point to a brain fuel deficit which appears to be specific to glucose, precedes cognitive decline associated with AD, and becomes more severe as MCI progresses toward AD. Since glucose is the brain’s main fuel, we suggest that gradual brain glucose exhaustion is contributing significantly to the onset or progression of AD. (iv) Interventions that raise ketone availability to the brain improve cognitive outcomes in both MCI and AD as well as in acute experimental hypoglycemia. Ketones are the brain’s main alternative fuel to glucose and brain ketone uptake is still normal in MCI and in early AD, which would help explain why ketogenic interventions improve some cognitive outcomes in MCI and AD. We suggest that the brain energy deficit needs to be overcome in order to successfully develop more effective therapeutics for AD. At present, oral ketogenic supplements are the most promising means of achieving this goal.

## Introduction

Compensating for deteriorating brain energy metabolism is the core feature of an emerging strategy aimed at delaying the onset and/or progression of AD. Relative to its size, the adult human brain requires a disproportionately large amount of energy that is provided principally by glucose. In those at risk of AD including cognitively healthy older people, regional brain glucose hypometabolism can be present long *before* the clinical diagnosis of AD. The brain’s alternative energy supply to glucose is unique compared to other organs in that it specifically requires ketones (also known as ketone bodies) to compensate for occasions when glucose supply to the brain is inadequate. In contrast to glucose, brain uptake of ketones appears to still be normal in AD. Hence, ketogenic interventions may help delay AD.

An AD treatment strategy focused on preventing brain energy starvation during aging is based on research that started at least 40 years ago. For instance, the unique dependence of the brain on ketones to replace low glucose supply has been known since the 1960s (Cahill, 2006). It has been proposed since the early 1980s that failing brain glucose supply to or metabolism by the brain could be contributing to AD risk or progression (Hoyer et al., 1988; Veech et al., 2001; Cunnane et al., 2011, 2016). Concrete clinical efforts to develop a ‘keto-neurotherapeutic’ strategy to bypass the problem with brain glucose metabolism in AD were first reported a decade ago (Reger et al., 2004; Henderson, 2008; Krikorian et al., 2012; Newport et al., 2015). Despite these pioneering studies, this approach to combat AD is still very much a novel area of research.

We see the challenge facing brain energy metabolism during aging through the lens of the similar challenge of assuring sufficient energy supply to the rapidly growing brain of the infant. At no time in our life cycle is the challenge of supplying the brain with sufficient energy more acute than in early human brain development. Indeed, the human species must have confronted this energy constraint for normal brain development when the brain started to triple in size more than 2 million years ago (Cunnane and Crawford, 2014). We suggest that the energetic (glucose) deficit confronted by the aging brain today is essentially the same as the challenge faced during brain expansion at the dawn of our species and that ketones were part of the solution then as now. Hence, it makes physiological sense to apply what we know about the importance of ketones in early brain development to the challenge of maintaining brain energy supply and brain function during aging.

This review will therefore focus on providing the rationale for proposing that deteriorating brain energy metabolism is a constraint for healthy cognitive aging that will have to be overcome in order to successfully limit the impact of AD no matter what therapeutic strategy is used (neurotransmitter-based, anti-amyloid, exercise, etc.). We will describe here our brain ketone PET studies in aging and AD with the ketone tracer, ^11^C-acetoacetate, because this tracer provides a valuable window on brain energy metabolism to compare with glucose. As we and others have previously proposed, the pre-symptomatic presence of brain glucose hypometabolism in people at risk of AD has clear implications for potential therapeutic strategies (Gibson et al., 2000; Veech et al., 2001; Blass, 2008; Henderson et al., 2009; Cunnane et al., 2011, 2016). We will also refer to a number of issues that will need to be addressed as this field matures.

## Deteriorating Brain Glucose Supply in Those At Risk of AD

**FIGURE 1:**
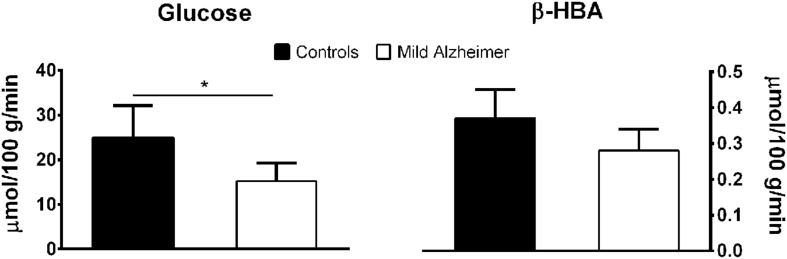
**Lower brain glucose uptake (mean ± SD; ^∗^*p* < 0.05) but normal brain β-HBA uptake measured by arterio-venous difference in mild AD compared to age-matched healthy controls.** These results are averaged from two previous publications (Lying-Tunell et al., 1981; Ogawa et al., 1996).

The concept that ketones could be of therapeutic value during brain aging hinges on demonstrating that there is a *pre-symptomatic* problem with brain glucose metabolism in people at risk of AD but who are still cognitively normal. Older people are a key focus of this work because they are at the highest risk of AD but this concept applies to any genetic, lifestyle, or demographic risk factor for AD. PET imaging with the glucose tracer, FDG, and oxygen tracer, ^15^O-oxygen, was first used to image brain energy metabolism in the late 1970s with reports on AD first appearing in the early 1980s (Benson et al., 1983). Since then, PET-FDG has been a cornerstone of human and animal studies on brain energy metabolism in aging and AD (Cunnane et al., 2011). Indeed, without PET-FDG, it is doubtful that brain glucose hypometabolism in AD would have become so widely studied because the only option besides PET, the arterio-venous difference method, is highly invasive and is used less and less in research today. Nevertheless, the arterio-venous difference method was the first to be used to assess brain oxygen and glucose uptake during aging (Dastur, 1985) and the first to show that specifically brain glucose uptake and not brain ketone uptake was significantly impaired in AD (Lying-Tunell et al., 1981; **Table 1** and **Figure 1**). The arterio-venous difference method also produced several ground-breaking reports comparing brain ketone and glucose uptake. These studies laid the foundation for our current understanding that ketones are an essential physiological brain fuel working in tandem with glucose to assure that brain energy requirements are being met on a daily basis, not just in infants or during starvation (Cunnane et al., 2016). The advantage of the arterio-venous difference method is that it permits quantification of global brain energy metabolism. Aside from its invasiveness, its disadvantage is that it provides no information about brain regions that are most or least affected in AD.

**Table 1:** Lower glucose consumption but not brain blood flow or oxygen consumption at the start of early-onset AD compared to healthy young adults or cognitively normal older adults (Hoyer et al., 1988; Hoyer, 1992).

	Young (*n* = 15)	Older (*n* = 11)	Start of early onset AD^∗^ (*n* = 20)
Cerebral blood flow (ml/100 g/min)	53 ± 5^¤^	56 ± 3	54 ± 3
Cerebral metabolic rate of O_2_ (ml/100 g/min)	3.5 ± 0.4	3.7 ± 0.5	3.4 ± 0.3
Cerebral metabolic rate of glucose (mg/100 g/min)	5.0 ± 0.8	5.0 ± 0.3	2.8 ± 0.3**


*^¤^Data are mean ± SD. ^∗^No brain atrophy on CT (no other measures of neuropathology available). ^∗∗^*p* < 0.05 vs. Older and Young groups.*

Decades of research with PET-FDG have made it abundantly clear that regional brain glucose uptake is defective in AD (Cunnane et al., 2011, 2016). This AD-type pattern is particularly evident in the parietal, posterior cingulate and temporal cortex and much less present in the occipital cortex and cerebellum. Indeed, the parieto-temporal pattern is relatively specific to AD itself and distinct from the pattern in other forms of dementia and from normal aging itself. This regional pattern of brain glucose hypometabolism in AD has generally been interpreted as a consequence of neuronal failure and death because, logically, brain regions with dysfunctional or dead brain cells need less fuel. However, this interpretation is insufficient because it does not take into account multiple examples of conditions in which the risk of AD is elevated and in which the regional brain glucose hypometabolism is present *before* the clinical (cognitive) onset of the disease. Examples of conditions in which regional brain glucose hypometabolism is present pre-symptomatically include carriers of the Pre-senilin-1 mutation, carriers of apolipoprotein E4, maternal family history of AD, cognitively healthy aging, and insulin resistance in both young and older persons (**Table 2**). Since regional brain glucose hypometabolism is present before measurable cognitive deficit in these conditions, it is clearly pre-symptomatic and therefore cannot only be a consequence of neuronal loss caused by AD. As such, it is plausible that brain glucose hypometabolism could increase the risk of developing AD. This argument has previously been made in detail (Cunnane et al., 2011, 2016) so we will focus here on two examples that collectively represent the populations at highest overall risk for AD – older people and those with insulin resistance (Ronnemaa et al., 2008; Craft, 2009, 2012; Matsuzaki et al., 2010; Schrijvers et al., 2010; Baker et al., 2011).

**Table 2:** Brain glucose hypometabolism in persons at risk of AD but in whom cognitive performance is normal.

	Mean age (y)	Brain region	Brain glucose hypometabolism (% difference from control)	Reference
Insulin resistant young women with PCOS	25	Frontal cortex	-9 to -14	Castellano et al., 2015a
		Middle temporal cortex		
Young adult carriers of Presenilin-1	30	Posterior cingulate	-14 to -25	Scholl et al., 2011
		Parietal cortex		
		Temporal cortex		
Young adult carriers of Apolipoprotein-E4	31	Parietal cortex	-9 to -11	Reiman et al., 2004
		Temporal cortex		
		Posterior cingulate		
		Prefrontal cortex		
Maternal family history of AD	43	Parietal cortex	-12 to -21	Mosconi et al., 2006
		Temporal cortex		
		Hippocampus		
		Entorhinal cortex		
		Posterior cingulate		
Cognitively healthy older adults	72	Frontal cortex	-10 to -18	Nugent et al., 2016
		Temporal cortex		
		Anterior cingulate		
		Putamen		
		Thalamus		
Pre-diabetic older persons	74	Temporal cortex	N/A	Baker et al., 2011
		Parietal cortex		
		Posterior cingulate		
		Precuneus		
		Prefrontal cortex		


*N/A, not available.*

## Deteriorating Brain Glucose Uptake During Aging

The risk of AD increases with advancing age but it has not been clear until recently whether healthy aging *per se*, i.e., cognitively normal and relatively free of overt risk factors for AD, is associated with deteriorating brain glucose uptake. This uncertainty has been due to a lack of a standard definition of healthy aging, a lack of sufficient verification in several reports as to whether or not cognition was normal in the older group, as well as infrequent quantification of brain glucose uptake to determine the *actual magnitude* of the problem (Nugent et al., 2014a). Knowing that there is a statistical difference in brain glucose across brain regions or between two groups is not sufficient; being able to assess the magnitude of the problem is essential in order to set a therapeutic target to counteract the problem.

**FIGURE 2:**
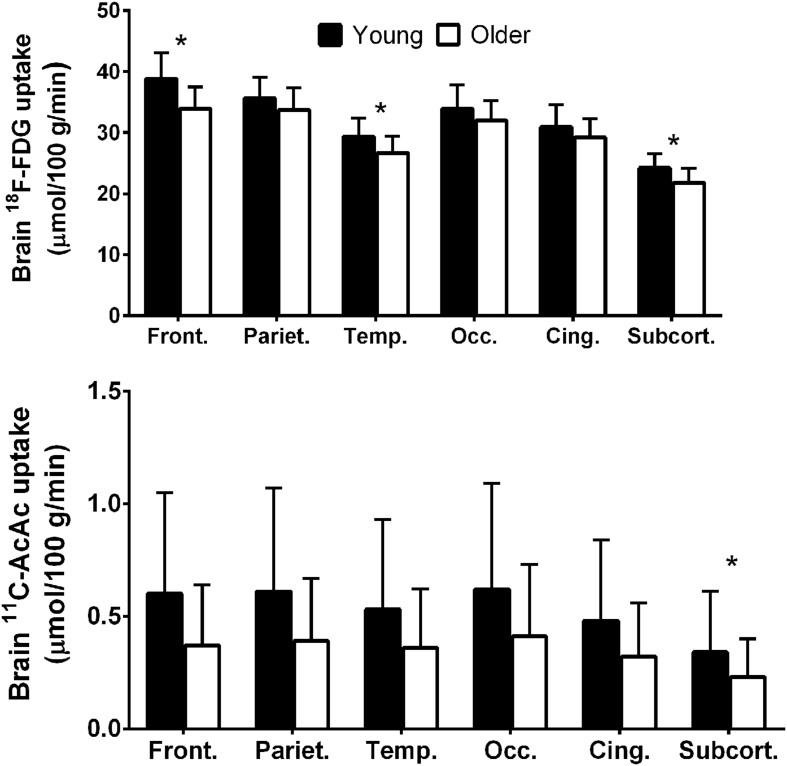
**Regional ^18^F-FDG (upper panel) and ^11^carbon-acetoacetate (^11^C-AcAc; lower panel) uptake in young adults (black bars; *n* = 30) compared to cognitively healthy older adults (white bars; *n* = 42; Nugent et al., 2014b, 2016).** Front., frontal lobe; Pariet., parietal lobe; Temp., temporal lobe; Occ., occipital lobe; Cing., cingulate gyrus; Subcort., subcortical regions. ^∗^*p* < 0.05.

We have therefore developed a database on brain ketone and glucose uptake in cohorts of cognitively normal young and older people. The older group had a minimum age of 65 years and was relatively free of overt disease. A detailed neuropsychological assessment showed that they were cognitively normal and had a metabolic profile as closely matched to healthy young adults as possible (**Tables 3** and **4**). Brain glucose uptake was quantified as CMRg with the units, μmol/100 g/min, both globally and regionally in each participant. This cognitively healthy older cohort had 9% lower global brain glucose uptake compared to our younger controls, a deficit that was mostly though not exclusively limited to the frontal cortex (-14%) and the caudate (-18%; Nugent et al., 2014a, 2016; **Figure 2**). This pattern is regionally different from the situation in AD where glucose hypometabolism is not only in the frontal cortex but includes glucose uptake that was as much as 33% lower in parts of the temporal and parietal cortex, 17% lower in the thalamus and 26% lower in the posterior cingulate cortex (Castellano et al., 2015b). Significantly lower glucose uptake in the frontal cortex is therefore commonly present in *cognitively healthy* older persons despite the absence of any clinical sign of AD.

**Table 3:** Demographics of our cognitively normal young and older adults (mean ± SD).

	Young	Older	*p*-value
Number	30	41	
Male/female	14/16	15/26	
Age (years)	26 ± 4	71 ± 5	≤0.001
Body Mass Index	23 ± 3	26 ± 4	0.001
Blood pressure (mm Hg)	114/69	133/79	0.001
Homocysteine (μM)	7.9 ± 1.5	10.1 ± 2.4	≤0.001
Hemoglobin A1c (%)	5.2 ± 0.2	5.8 ± 0.3	≤0.001
Glucose (mM)	4.9 ± 0.4	5.0 ± 0.5	
Acetoacetate (μM)	162 ± 129	129 ± 107	
β-Hydroxybutyrate (μM)	351 ± 298	272 ± 259	


*Their cognitive scores are reported in **Table 4** and their brain glucose and ketone uptake in **Figure 2**.*

**Table 4:** Cognitive scores (mean ± SD) of healthy young and older adults reported in **Figure 2** and **Table 3**^∗^.

	Young	Older	*p*-value
**Global cognition**			
MMSE	29.9 ± 0.3	29.4 ± 0.9	0.051
**Speed processing and attention**			
Digit symbol substitution	11.4 ± 2.5	10.9 ± 2.3	0.875
**Executive function**			
Trail making number sequencing	12.5 ± 1.7	11.0 ± 3.3	0.247
Trail making number-letter switching	12.0 ± 1.4	10.4 ± 3.0	0.226
Stroop-inhibition	12.1 ± 2.5	10.6 ± 2.7	0.096
Stoop-inhibition/switching	10.5 ± 2.9	10.4 ± 2.3	0.999
Verbal fluency-letter	10.1 ± 2.9	9.9 ± 3.3	0.968
Verbal fluency-category	12.7 ± 3.1	11.5 ± 2.9	0.397
**Working memory**			
Digit span	9.2 ± 2.6	7.9 ± 3.0	0.555
Spatial span	11.8 ± 3.3	11.4 ± 2.8	0.666
**Episodic memory**			
RCFT-Immediate recall	61.3 ± 15.4	67.3 ± 12.8	0.005
RCFT-Delayed recall	62.2 ± 10.7	69.0 ± 11.8	≤0.001
VPA-Immediate recall	12.3 ± 2.5	12.4 ± 3.3	0.437
VPA-Delayed recall	12.1 ± 1.0	12.7 ± 2.6	0.017
LM-Immediate recall	14.9 ± 2.0	13.2 ± 3.0	0.289
LM-Delayed recall	15.9 ± 1.8	14.0 ± 2.7	0.170


*^∗^Results corrected for age and education. MMSE, mini-mental state examination; RCFT, Rey complex figure test; VPA, verbal paired associates; LM, logical memory.*

## Deteriorating Brain Glucose Uptake in Insulin Resistance

The metabolic profile of the older cohort described above was moderately well matched to the younger group but some parameters did differ significantly, i.e., body-mass index, blood pressure, homocysteine, and some measures of glucose homeostasis were not identical and could have adversely influenced brain glucose metabolism (Nugent et al., 2016; **Table 3**). However, it is difficult to find older people in whom the metabolic profile is strictly within the same limits as adults <35 years-old. Mild insulin resistance seems to be a common feature of aging, but it is by no means limited to the older population. The importance of this point became clear to us when we studied young women with mild insulin resistance due to PCOS. They had a brain glucose uptake deficit in the superior and middle frontal cortex of about 14%, i.e., a profile resembling that of people in their 70 and 80s (Castellano et al., 2015a). PCOS is a multi-factorial endocrine disease involving not only mild-moderate insulin resistance but also infertility and hyperandrogenism. The mild insulin resistance was of particular interest to us because it is associated with increased risk of AD in middle-aged and older adults (Ronnemaa et al., 2008; Craft, 2009, 2012; Matsuzaki et al., 2010; Schrijvers et al., 2010; Baker et al., 2011).

In our women with PCOS, glucose uptake in several brain regions was significantly inversely correlated to both the mild insulin resistance and to fasting plasma glucose. Recent studies in young women with PCOS demonstrate that insulin resistance is associated not only with impaired brain glucose metabolism but also with altered white matter microstructure and cognitive performance (especially working memory) in young adults (Castellano et al., 2015a; Rees et al., 2016). These results suggest that glucose dysregulation and the development of a pattern of deteriorating brain glucose in older people can start in the second to third decade of life (Burns et al., 2013; Ishibashi et al., 2015). Whether women with PCOS are predisposed to a higher risk of cognitive decline as they age and whether this apparently increased risk of cognitive decline can be prevented or reversed requires further attention.

**FIGURE 3:**
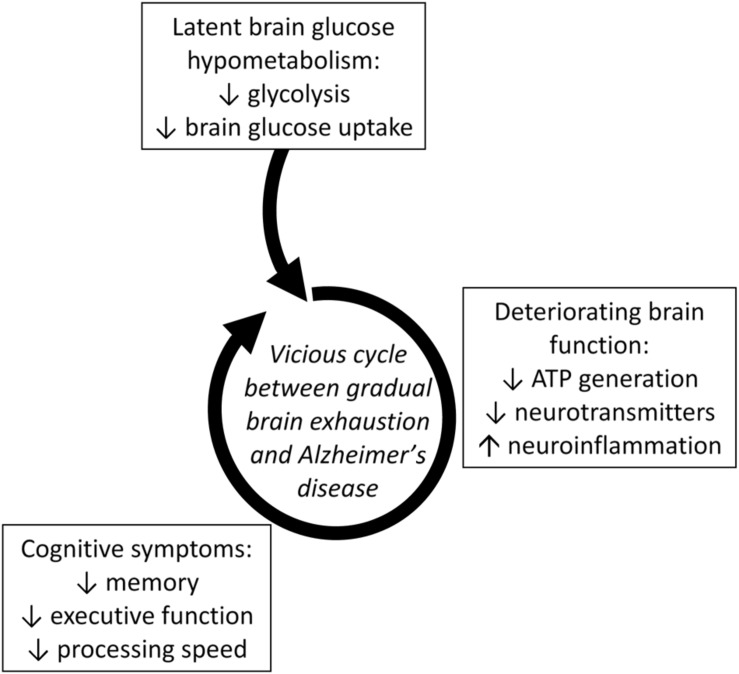
**Latent brain glucose hypometabolism leading to a vicious cycle of accelerating metabolic deterioration, neuroinflammation and neuronal dysfunction that increases the risk of developing AD (Cunnane et al., 2016).** Lower brain glucose uptake is a direct reflection of lower glycolysis in brain cells. Lower glycolysis contributes to impaired brain function (ATP generation and neurotransmitter production) which in turn contributes to cognitive symptoms. Deteriorating cognitive function then reduces demand for glucose turning this sequence into a vicious cycle.

Thus, regional brain glucose hypometabolism can be present in those at risk of AD due to old age, or to insulin resistance regardless of age. Multiple mechanisms are undoubtedly involved in the mechanism by which insulin resistance affects the onset and/or progression of AD (Schioth et al., 2012). This pre-symptomatic glucose uptake deficit is commonly but not exclusively in the frontal cortex and its magnitude is of the order of 12–15% (Cunnane et al., 2011; Castellano et al., 2015b; Nugent et al., 2016). We interpret these findings to mean that a vicious cycle can develop in which chronic pre-symptomatic brain glucose hypometabolism develops and then contributes to deteriorating neuronal function, further decline in demand for glucose, and the emergence of cognitive decline which then further decreases brain glucose consumption (**Figure 3**; Cunnane et al., 2011, 2016). Chronic sedentarity commonly contributes to chronic hyperinsulinemia and insulin resistance which not only compromise tissue glucose uptake but also decreased ketogenesis and ketone metabolism (Fukao et al., 2004; Bickerton et al., 2008). In effect, this puts the aging brain at risk of exhaustion because now it is not only getting insufficient glucose but is also less ketones (Cunnane et al., 2011, 2016; Mamelak, 2012).

## Ketones: Essential in Early Human Brain Development

The adult brain accounts for about 2% of adult body weight but it consumes about 20–23% of the energy needs of the whole body. Like other organs, the brain has a back-up fuel for occasions when glucose supply is insufficient, i.e., during fasting, starvation, strenuous exercise, or malnutrition. Ketones are the only significant alternative fuel to glucose for the brain, which is a unique situation because as far as is known all other organs use free fatty acids to replace insufficient availability of glucose (Cunnane et al., 2011). β-HBA and AcAc are the two ketones that replace glucose for the brain. The third ketone, acetone, is a decarboxylation product of AcAc that is mainly excreted on breath but can also potentially enter intermediary metabolism. The focus here will be on β-HBA and AcAc; neither acetone nor the less common five carbon ketones derived from odd-chain fatty acids will be discussed further.

Unlike in the human adult in whom the brain appears to use ketones only to compensate for periodic insufficiency in glucose supply, in the developing infant, ketones are *essential* both as a major fuel and also as the main substrate for brain lipid synthesis (Cunnane et al., 2003, 2016). Ketones are an essential fuel for the human neonatal brain because there is insufficient glucose available to meet its brain energy requirements (Settergren et al., 1976; Robinson and Williamson, 1980; Bougneres et al., 1986). This important role of ketones in infant brain development and energy metabolism starts to develop in the fetus (Adam et al., 1975). Postnatally, the brain’s dependence on ketones is made possible because infants are normally in a *sustained state of mild ketosis* (0.2–0.5 mM β-HBA). This neonatal ketosis is present regardless of whether the infant has just been fed or is in a post-prandial state, i.e., the ketosis is not a function of food restriction or hypoglycemia (Settergren et al., 1976). This contrasts with the adult human in whom 0.5 mM β-HBA in plasma is normally only achieved after 24–48 h fasting accompanied by hypoglycemia and hypoinsulinemia.

The constant state of ketosis in infants is due mostly to MCFAs supplied in breast milk; indeed, the milk of most (probably all) mammalian species contains 10–20% of all fatty acids as MCFA (Hilditch and Meara, 1944; Insull and Ahrens, 1959; Breckenridge and Kuksis, 1967). Some of the MCFA in breast milk end up in the adipose stores of the infant and can be used days or weeks later, thereby in effect extending lactation for some period of time with respect to the availability of ready-made ketone substrates (Sarda et al., 1987). However, unlike in humans, the offspring of other terrestrial mammals have virtually no adipose tissue so they have very limited ability to store MCFA and, hence, poor ability to generate ketones post-weaning (Robinson and Williamson, 1980). Human babies on the other hand have significant subcutaneous fat stores, i.e., 500–600 g if they are born at term, but markedly less if they are born pre-term. After lactation ends, the long chain fatty acids and the small amount of MCFA stored in adipose tissue provide the substrate to prolong mild ketonemia for many months. Incidentally, in addition to the liver, the infant gut can also synthesize ketones (Bekesi and Williamson, 1990).

## Ketones: The Brain’s Preferred Fuel

That ketones are the main reserve fuel for the adult human brain when glucose supply is compromised by starvation was convincingly demonstrated in the now classic studies of medically supervised long-term starvation reported by Owen et al. (1967) and Drenick et al. (1972). The brain’s need for energy during prolonged starvation can be met by the high ketogenic capacity of the liver which can produce up to 150 g ketones/day (Flatt, 1972; Reichard et al., 1974). Despite the liver’s high energy consumption, it cannot catabolize ketones, so they diffuse into the circulation where they become available to all organs. However, as starvation progresses, other organs, particularly skeletal muscle, come to use free fatty acids more efficiently so ketones therefore become increasingly available for the brain which has no other energy substrate to replace low glucose (Owen and Reichard, 1971; Drenick et al., 1972).

In adults, long chain fatty acids stored in adipose tissue are the main substrate for ketogenesis. They are released as free fatty acids when low blood glucose in turn causes hypoinsulinemia (Mitchell et al., 1995). Free fatty acids entering the liver are beta-oxidized, generating acetyl-CoA. As hypoinsulinemia continues, free fatty acid delivery to the liver continues and acetyl-CoA starts to accumulate because its concentration exceeds the capacity of the citric acid cycle to metabolize it. Acetyl-CoA accumulation in the liver leads to condensation of two acetyl-CoAs to ketones via hydroxyl-methyl-glutaryl CoA. During short-term fasting, ketone metabolism generally matches ketone synthesis so plasma ketones usually do not rise much above ≤0.3 mM (Hall et al., 1984; Balasse and Fery, 1989; Avogaro et al., 1990; **Table 5**). Greatly increased ketogenesis relative to ketone clearance after 3–5 days fasting causes plasma ketones to rise about 10-fold. In the presence of hypoglycemia, the liver depends on gluconeogenesis to support the energy costs of ketogenesis (Flatt, 1972; Garber et al., 1974).

**Table 5:** Overview of ketones (β-hydroxybutyrate + acetoacetate) kinetics in humans.

	Fasting period	Plasma ketones (mM)	Utilization (μmol/kg/min)	Synthesis (μmol/kg/min)	Metabolic clearance (ml/kg/min)	Urinary excretion (μmol/min)
Healthy adults	12–16 h^A,B,C^	0.1–0.3^∗^	3–5	2–5	18	ND
	3 days^D^	2.5	ND	10	ND	4
Healthy adults + 30 min exercise	16 h^B,E^	0.2–0.4	6	6	21	ND
	3–5 days^B,E,F^	4–5	20	22	4–6	ND


*^∗^Mean values extracted from the references indicated. ND, no data available. **^A^**Hall et al., 1984; **^B^**Fery and Balasse, 1983; **^C^**Avogaro et al., 1990; **^D^**Garber et al., 1974; **^E^**Fery and Balasse, 1986; **^F^**Balasse et al., 1978.*

**FIGURE 4:**
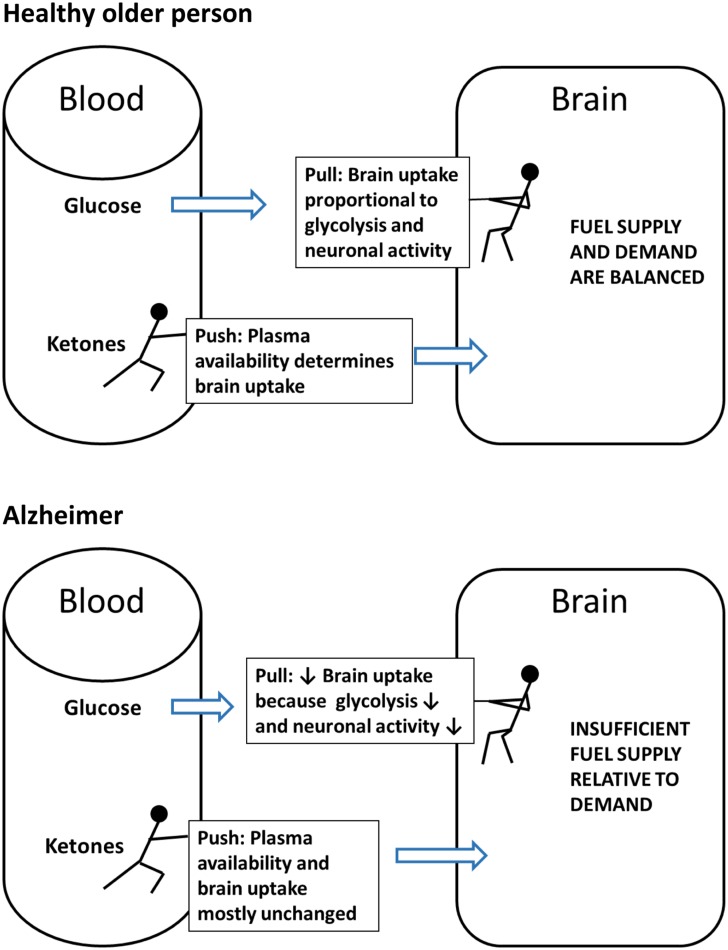
**The contrasting ‘Push-Pull’ mechanism of brain fuel supply.** Glucose is pulled from the blood into the brain as a function of the brain’s metabolic demand during neuronal activation. Under normal conditions (excluding insulin resistance), ketones are pushed from the blood into the brain in direct proportion to their plasma concentration and irrespective of plasma glucose level.

Ketone transport into tissues including the brain occurs via monocarboxylic acid transporters of which there are several sub-types (Simpson et al., 2007). Monocarboxylic acid transporter expression in the brain responds rapidly to hyperketonemia (Halestrap and Price, 1999; Pan et al., 2001). Thus, brain uptake of ketones is normally directly proportional to their plasma concentration over at least the range of 0.02–12 mM (Cunnane et al., 2011, 2016; Courchesne-Loyer et al., 2013). In contrast to ketones which are ‘pushed’ into the brain in proportion to their plasma concentration, glucose is ‘pulled’ into the brain in proportion to its utilization by astrocytes and neurons (**Figure 4**). The ‘push-pull’ strategy assures that ketones will enter the brain under conditions in which glucose availability is decreased and ketone synthesis is stimulated.

Both short-term PET and arterio-venous difference studies in humans show that brain glucose consumption *decreases* as ketone availability to the brain increases (Hasselbalch et al., 1995). These results suggest that ketones are actually the preferred energy substrate for the brain because they enter the brain in proportion to their plasma concentration irrespective of glucose availability; if the energy needs of the brain are being increasingly met by ketones, glucose uptake decreases accordingly. This decrease in brain glucose uptake when both ketones and glucose are available supports the notion that ketones are the brain’s preferred fuel. Nevertheless, it is uncommon for both ketones and glucose to be available; normally, when one is increased in the blood the other is decreased. Under conditions of normal energy sufficiency and three meals per day, ketogenesis is supressed and glucose supplies >95% of the brain’s energy requirements; hence, glucose (or fuels derived from glucose, i.e., lactate or pyruvate) is the brain’s dominant but not actually its preferred fuel.

The problem for the aging brain is that low glucose *supply* in the blood is not the same as low brain glucose *utilization*. When blood glucose decreases, ketogenesis normally occurs rapidly in response to decreased insulin. However, when brain glucose utilization is decreased, plasma insulin does not necessarily decrease; indeed, during aging, plasma insulin and glucose are commonly mildly elevated and there is a state of mild-moderate insulin resistance. Hyperinsulinemia inhibits the normal ketogenic response (Bickerton et al., 2008), thereby putting the aging brain in double jeopardy of being deprived of both its primary fuels. We believe that this problem is at the root of the vicious cycle between deteriorating brain fuel uptake/availability and deteriorating brain function that leads to AD.

## Ketosis, MCT, and Brain Function

In discussing keto-neurotherapeutics, it is essential to distinguish between nutritional or dietary ketosis and pathological ketosis; they differ in origin, in severity of ketosis and in medical consequences. Nutritional ketosis is a physiological response to sustained low carbohydrate intake resulting in low plasma glucose and insulin, and plasma ketones of 2–5 mM after a week or so. Nutritional ketosis has never been shown to induce ketoacidosis, i.e., to alter acid-base balance or to lower blood pH whether after experimental ketone infusion (Hasselbalch et al., 1995) or during medically supervised starvation lasting as long as 60 days (Drenick et al., 1972). Nutritional ketosis can be sustained for weeks, months, or even years; indeed, there are numerous metabolic and cardiovascular benefits of nutritional ketosis in addition to the clinical benefits that are well-documented for intractable epilepsy. Experimental ketone infusion shows that nutritional ketosis is usually self-limiting because raising ketones to about 3–5 mM by sodium-AcAc infusion stimulates insulin secretion which in turn rapidly reduces plasma ketones (Owen et al., 1973). Experimental insulin infusion can be used to induce severe hypoglycemia but in the presence of prolonged starvation does not induce ketoacidosis (Drenick et al., 1972).

Pathological ketoacidosis on the other hand is a medical emergency arising in type 1 diabetes because of acute severe insulin deficiency due usually to interruption of insulin injection. Plasma ketones generally exceed 10–15 mM and blood pH may decrease to 7. Comorbidities such as alcoholism, malnutrition and/or serious infection often contribute to exacerbating insulin deficiency and increase the severity of the ketoacidosis. Hence, unlike pathological ketosis, nutritional ketosis is a safe and sustainable condition in which insulin decreases due to low carbohydrate intake not because of a disease process. It can be undertaken with medical supervision and usually involves low to negligible risk to the individual. Nutritional ketosis can also be rapidly reversed by consuming carbohydrate.

**FIGURE 5:**
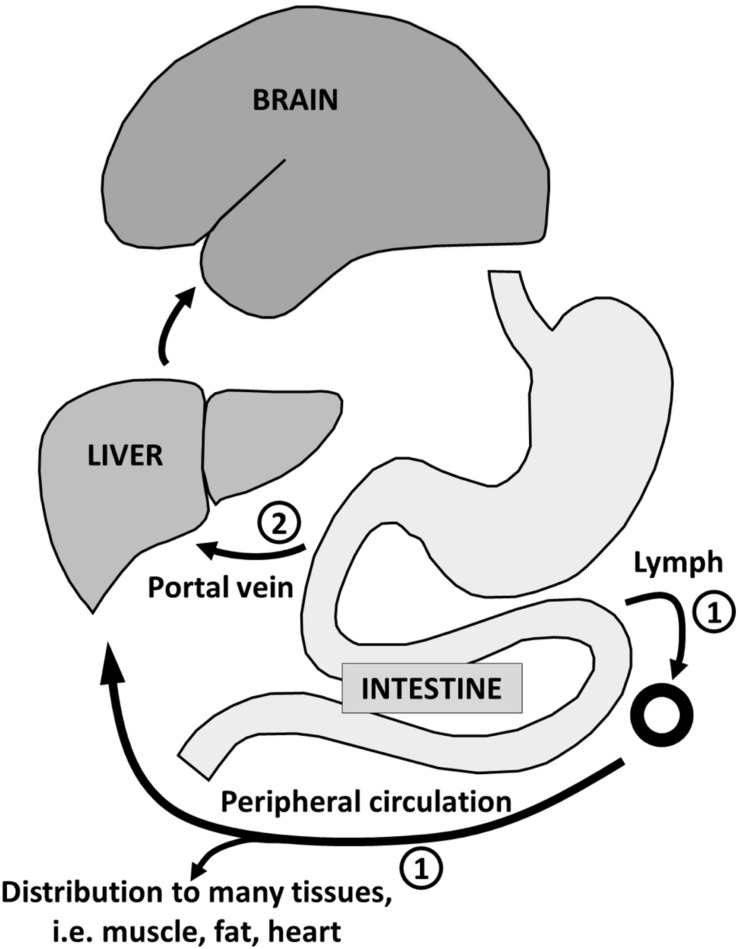
**Absorption of long-chain fatty acids ➀ takes place in the intestine with transport *via* chylomicrons and the lymphatic system to the peripheral circulation with distribution to adipose tissue and other organs (except the brain).** The route for medium-chain fatty acid absorption ➁ is via the portal vein directly to the liver where they are almost entirely beta-oxidized. Some of the resulting acetyl-CoA is transformed into ketones which reenter the circulation for use as energy substrate principally by the brain.

Long chain fatty acids (14–22 carbons) stored in adipose tissue are normally the main substrate for ketogenesis in adults because the diet rarely contains MCFA. However, breast-feeding infants are in mild ketosis principally because of the MCFA they are consuming from breast milk (Cunnane and Crawford, 2014). There are two reasons why MCFA are particularly effective ketogenic substrates (**Figure 5**): first, an oral dose of MCFA is mostly absorbed from the gut directly into the portal vein. This is a more rapid route to the liver than for dietary long chain fatty acids which are absorbed as chylomicrons via the lymphatic system and pass into the peripheral circulation before reaching the liver. Second, unlike a long chain fatty acid which requires carnitine-dependant activation to a Coenzyme A before accessing the mitochondria, beta-oxidation of MCFA occurs without activation by carnitine. The net result is more rapid beta-oxidation and ketogenesis of MCFA than from long chain fatty acids (Guillot et al., 1993).

The potential clinical benefit of MCTs as a rapid energy source was initially reported for surgically stressed and/or malnourished patients (Bach and Babayan, 1982; De Gaetano et al., 1994; St-Onge and Jones, 2002). The ketogenic effect of MCT was already well-known (Freund and Weinsier, 1966; Bach and Babayan, 1982) but was not clinically exploited until they were tried as an alternative ketogenic approach to the ketogenic diet in refractory childhood epilepsy (Huttenlocher, 1976). At about the same time, MCT started to be introduced into formula milk for infants. Owing to the interest in ketones as possible brain fuels to bypass deteriorating brain glucose, the effects of MCT supplementation on cognitive outcomes has been investigated in mild-moderate AD in studies lasting several months (Henderson et al., 2009) but also after just a single dose of MCT (Reger et al., 2004). Very high fat diets are also ketogenic by virtue of their low carbohydrate content and have been reported to have beneficial effects on cognitive and cardiovascular outcomes in mild cognitive impairment (MCI), the prodromal state to AD (Krikorian et al., 2012). In Type 1 diabetics undergoing controlled experimental hypoglycemia caused by insulin infusion, MCT improve several cognitive outcomes (Page et al., 2009). Collectively, these reports suggest that beyond the well-established role of MCFA in infant development and the use of MCT in parenteral and enteral nutrition and intractable epilepsy, several aspects of cognitive function that deteriorate during acute hyperinsulinemia or with cognitive decline associated with AD can be partially or completely normalized when ketones contribute to fuelling the brain (**Table 6**).

**Table 6:** Clinical studies in which hormonal and cognitive responses indicate that ketones maintain brain function by compensating for hypoglycemia.

	Treatment	Reference
**Acute studies in healthy adults**		
Controlled insulin-induced hypoglycemia ± fasting in obesity (*n* = 9)	Treatment: 2 h insulin infusion ± 60 days fast	Drenick et al., 1972
	Outcomes: ↓ effect of acute severe hypoglycemia (0.5 mM in one case), including ↓ mental confusion, anxiety, sweating, tachycardia, blood pressure if fasted for 60 days before the insulin infusion	
Controlled insulin-induced hypoglycemia in healthy adults (*n* = 6)	Treatment: 4 h i.v. β-HBA infusion	Amiel et al., 1991
	Dose: 30 μmol/min/kg body weight	
	Outcomes: ↓ hormonal response to hypoglycemia	
Controlled insulin-induced hypoglycemia in healthy adults (*n* = 13)	Treatment: 6 h i.v. β-HBA infusion	Veneman et al., 1994
	Dose: 20 μmol/min/kg body weight	
	Outcomes: ↓ hormonal and cognitive symptoms of acute hypoglycemia	
Controlled insulin-induced hypoglycemia in type 1 diabetes (*n* = 11)	Treatment: oral MCT	Page et al., 2009
	Dose: 40 g in three stages (20, 10, 10 g)	
	Outcomes: ↓ cognitive symptoms of acute hypoglycemia	
**Age-associated cognitive decline**		
Mild cognitive impairment (*n* = 23)	Treatment: 6 weeks high fat ketogenic diet	Krikorian et al., 2012
	Outcomes: ↑ secondary memory performance	
Mild-moderate AD (*n* = 20)	Treatment: single dose of 95% octanoate	Reger et al., 2004
	Dose: 40 g orally	
	Outcomes: ↑ cognitive score in Apolipoprotein E4(-) patients	
Mild-moderate AD (*n* = 77)	Treatment: 90 days 95% octanoate	Henderson et al., 2009
	Dose: 20 g/d orally	
	Outcomes: ↑ cognitive score in Apolipoprotein E4(-) patients	
Severe AD (*n* = 1)	Treatment: 20 months MCT + coconut oil (4:3), including ketone ester	Newport et al., 2015
	Dose: 165 ml/d orally	
	Outcomes: ↑ mood, affect, self-care, and cognitive and daily activities	


Salts or esters of AcAc and β-HBA can also be directly administered orally or by intra-venous infusion (Hasselbalch et al., 1996; Plecko et al., 2002; Clarke et al., 2012), and inhibit the autonomic and neurological symptoms of acute severe experimental hypoglycemia in humans (Amiel et al., 1991; Veneman et al., 1994). The safety of sustained oral use of a β-HBA-monoester and its anecdotal utility in improving some aspects of cognitive function in an advanced case of early onset AD have recently been reported (Newport et al., 2015) and are being increasingly investigated in animal models (D’Agostino et al., 2013; Viggiano et al., 2015).

## MCT: A Safe, Efficient Ketogenic Substrate Across the Lifespan

**FIGURE 6:**
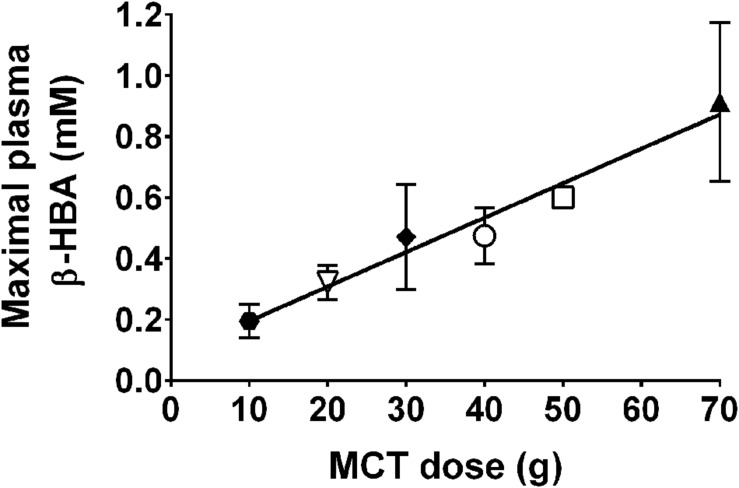
**Direct relationship between oral dose of MCTs and maximal observed plasma β-HBA (*r* = 0.95; *p* < 0.0001).** MCT were given as a single dose (Seaton et al., 1986; Pi-Sunyer et al., 1969; Reger et al., 2004; Page et al., 2009) or multiple doses (Henderson et al., 2009) for days to weeks. Data for the same dose are pooled from multiple sources: 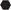 10 g MCT; (Henderson et al., 2009). 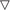 20 g MCT; (Henderson et al., 2009). 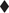 30 g MCT. 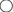 40 g MCT; (Reger et al., 2004; Page et al., 2009). 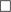 50 g MCT; (Seaton et al., 1986). 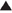 70 g MCT; (Pi-Sunyer et al., 1969; Freemantle et al., 2009).

There is normally no further opportunity to consume MCFA once breast-feeding is terminated. However, coconut and palm kernel oils contain MCFA. The MCFA-enriched fraction of these ‘tropical’ oils can be concentrated resulting in a generic MCT product containing mostly fatty acids of eight (octanoic or caprylic acid) and 10 carbons (decanoic or capric acid). The ratio of caprylic and capric acids and their proportion of the total can vary widely from one MCT product to another. Notwithstanding the generic nature of MCT and different study designs to assess their metabolism, there is a significant positive correlation between the oral dose of MCT taken and the maximal plasma β-HBA level achieved (**Figure 6**). This dose-response relationship can be used to estimate a therapeutic dose of MCT needed to achieve particular plasma ketone level. Octanoic acid can be taken up by the brain (Kuge et al., 1995; Ebert et al., 2003) so it may have direct effects on brain function including but not limited to conversion to ketones by astrocytes (Auestad et al., 1991).

Healthy older people have the same plasma ketone response and beta-oxidize ^13^C-β-HBA to ^13^C-CO_2_ to the same extent after a standard high fat ketogenic breakfast containing 70 g MCT as middle aged or young adults (76 years-old vs. 50 or 23 years-old, respectively; Freemantle et al., 2009). Another report suggests that the plasma ketone response to 18 h fasting is somewhat higher in older compared to younger adults (London et al., 1986). However, in our experience, plasma β-HBA and AcAc tend to be lower after an overnight fast in cognitively healthy older vs. young adults but so far the trend is not significant owing to wide inter-individual variability in plasma ketone data (Nugent et al., 2014b, 2016; **Table 2**). Hence, it seems likely that the capacity to produce and utilize ketones does not change appreciably during healthy aging but this still requires further work.

Medium chain triglyceride are saturated fats and, as such, their consumption is commonly associated with increased cardiovascular risk. However, consuming 30 g/d of MCT for 30 days does not adversely affect serum glucose, insulin, triglycerides, cholesterol, free fatty acids, body weight, or body-mass index (Courchesne-Loyer et al., 2013). In extensive tests, the safety of oral MCT at up to 1 g/kg/day is well-established in all species including humans (Bach and Babayan, 1982; Traul et al., 2000). MCT have important uses in parenteral nutrition and are widely present in infant formula. Nevertheless, they can have secondary side effects involving gastrointestinal distress, gastric reflux, and possible diarrhea, issues that can usually be mitigated by gradual dose titration.

## Regional Brain Ketone Uptake in AD

The aforementioned clinical reports of a cognitive benefit of MCT in AD (Reger et al., 2004; Henderson, 2008; Krikorian et al., 2012; Newport et al., 2015) are still preliminary and require replication on a larger scale. Nevertheless, they provisionally support the hypothesis that a regional brain glucose deficit contributes to impaired cognition associated with aging and that this deficit can at least in part be bypassed by ketogenic treatments. A core element of this interpretation is that brain cells and/or networks that were previously dysfunctional can start to function more normally again once they are provided with more fuel, i.e., they were starving or exhausting but not dead; otherwise this cognitive improvement would not be possible.

**FIGURE 7:**
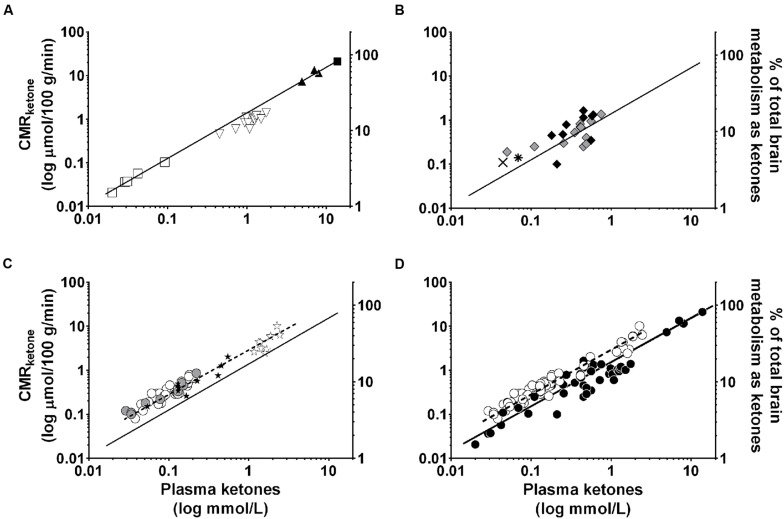
**Direct, linear relation between plasma ketone concentration (X axis), brain ketone uptake (left-hand Y axis), and percent contribution by ketones to total brain energy requirement (right-hand Y axis).** Data are for adults. Two relationships are shown, one for plasma β-HBA vs. the rate of brain β-HBA uptake (solid line, *R*^2^ = 0.97; *Y* = 1.57X – 0.20; *p* < 0.0001; **A–D**), and the other for plasma acetoacetate vs. the rate of brain acetoacetate uptake (Castellano et al., 2015b; dotted line, *R*^2^ = 0.83; *Y* = 3.46X – 0.03; *p* < 0.0001; **C,D**). Units are the same for both ketones – CMR (μmol/100 g/min). The data have been combined from several sources. **(A)** Plasma β-HBA vs. brain uptake in post-prandial state (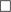) (Blomqvist et al., 1995), after β-HBA infusion (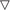) (Blomqvist et al., 2002), as well as after a 40 day fast (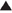) (Owen et al., 1967), or 60 day fast (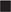) (Drenick et al., 1972). **(B)** Two studies of plasma β-HBA vs. brain uptake in AD [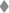] and healthy older controls (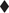) (Lying-Tunell et al., 1981), and AD (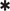) and healthy older controls (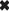) (Ogawa et al., 1996). **(C)** Plasma acetoacetate vs. brain uptake in AD (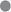) and cognitively healthy age-matched controls (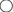) (Castellano et al., 2015b), as well as before (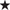) and 4 days after (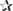) a very high fat ketogenic diet in healthy adults (Courchesne-Loyer et al., unpublished). **(D)** Pooled data from **(A–C)**. All the brain β-HBA uptake data are from arteriovenous difference studies except for the one report which used ^11^C-β-HBA PET (Blomqvist et al., 1995). The brain acetoacetate uptake data were obtained using ^11^C-acetoacetate PET (Castellano et al., 2015b). Each symbol represents whole brain ketone uptake in a single individual except when not available in the original publication, i.e., Drenick et al. (1972) for which 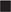 represents the mean of *n* = 5 participants, and Ogawa et al. (1996) for which 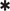 and 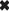 both represent the mean of *n* = 7. The relationship between plasma β-HBA and the percent of brain energy consumption supplied by β-HBA in adults is broadly as follows: at plasma β-HBA values around 0.1 mM, ketones supply >5% of brain energy; at 1 mM β-HBA, they supply about 10–15%; at 5–7 mM β-HBA, 50–65% and over 7–8 mM β-HBA, >75% of brain energy consumption. For a given plasma acetoacetate concentration, acetoacetate is taken up by the brain more rapidly than β-HBA which explains why the dotted regression line for acetoacetate lies above that of the solid line for β-HBA.

Apart from needing further cognitive studies, a crucial step in building the case that ketones could have beneficial neurotherapeutic properties is to be able to measure brain uptake of ketones in disease and before and after ketogenic interventions. We developed a PET research program using the ketone tracer, ^11^C-AcAc, to better understand the relation between brain fuel uptake and brain function in people with or at risk of AD. We use this dual tracer PET protocol to compare the brain uptake of ^11^C-AcAc to that of FDG in each individual studied. We quantify the magnitude of FDG and ^11^C-AcAc uptake regionally throughout the brain. The kinetics of brain ketone metabolism assessed using PET or arterio-venous difference suggest a one-tissue compartment model in which brain utilization essentially matches brain uptake (Lying-Tunell et al., 1981; Blomqvist et al., 1995; Ogawa et al., 1996). PET studies of brain ^11^C-β-HBA uptake in humans have already confirmed the earlier arterio-venous difference studies showing that the brain uptake of β-HBA is directly proportional to plasma β-HBA over a wide range of plasma ketone concentrations 20 μM to >10 mM (Lying-Tunell et al., 1981; Blomqvist et al., 1995, 2002; **Figure 7**). However, prior to our work no one had established whether the slope of the brain/plasma ketone relationship changed during aging or AD, or after taking a ketogenic supplement.

**FIGURE 8:**
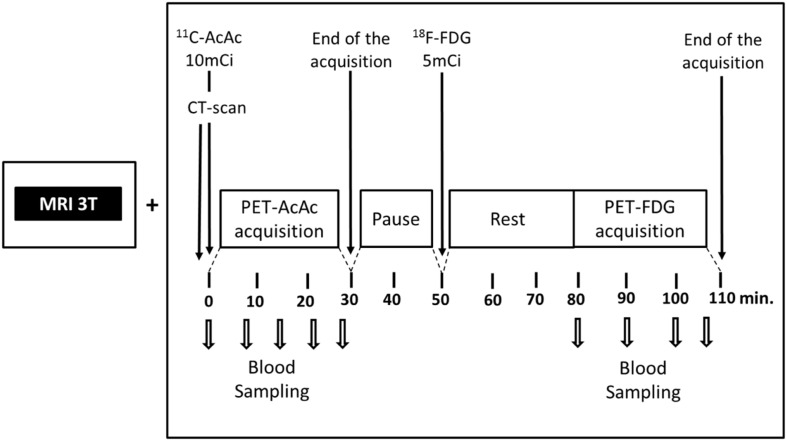
**Our ^11^carbon-acetoacetate (^11^C-AcAc) and ^18^F-FDG PET protocol (Pifferi et al., 2011; Roy et al., 2012; Nugent et al., 2014b; Castellano et al., 2015b).** The MRI is used for regional segmentation during the PET image analysis.

Acetoacetate is the ketone that actually enters the mitochondria and is catabolized to acetyl CoA. Since synthesis of ^11^C-AcAc is easier than for ^11^C-β-HBA (Tremblay et al., 2007), we chose ^11^C-AcAc as our brain ketone PET tracer. In our PET protocol, ^11^C-AcAc is infused first followed by a wash-out period and then FDG is infused (**Figure 8**). A period of time equivalent to four half-lives of ^11^C occurs between the ^11^C-AcAc infusion and the acquisition of the FDG image. This dual tracer technique allows for a quantitative same-day comparison of brain uptake of glucose and ketones thereby avoiding the unnecessary inconvenience to the participant of returning a second time, as well as reducing the biological variability between PET scans done on different days. This dual tracer protocol has been applied in human (Nugent et al., 2014b; Castellano et al., 2015b) and animal studies (Pifferi et al., 2011; Roy et al., 2012).

**FIGURE 9:**
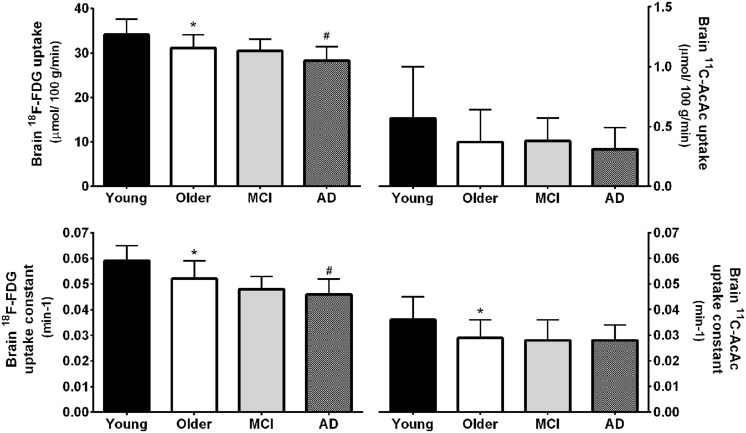
**(Upper)** Whole brain ^18^F-FDG uptake was 9% lower in mild AD (*n* = 12) compared to cognitively normal age-matched controls (Older; *n* = 42; #*p* < 0.05 corrected for false discovery rate). ^18^F-FDG uptake was also 9% lower in healthy older adults compared to younger adults (Young; *n* = 30; ^∗^*p* < 0.05 corrected for false discovery rate). Values for mild cognitive impairment (MCI; *n* = 9) were not statistically different between the Older and AD groups. In contrast to FDG, whole brain ^11^carbon-acetoacetate (^11^C-AcAc) uptake did not differ significantly between any groups. **(Lower)** statistical differences were present for the ^18^F-FDG and ^11^C-AcAc uptake constants (Nugent et al., 2014b; Castellano et al., 2015b).

We have compared brain uptake of FDG and ^11^C-AcAc in early AD (Castellano et al., 2015b; **Figure 9**). This study had three aims: first, to confirm for the first time using PET methodology the arterio-venous difference reports of normal ketone but low brain glucose uptake early in AD (Ogawa et al., 1996). Second, to assess brain fuel metabolism early in AD rather than the more advanced stages previously reported (Lying-Tunell et al., 1981). Third, to quantify the *regional* pattern of brain uptake of both fuels under post-prandial conditions, information that arterio-venous difference studies cannot provide. Our FDG and ^11^C-AcAc PET studies have so far confirmed several important points: first, as reported previously by arterio-venous difference in the brain taken as a whole (**Figure 1**), global CMRg was 14% lower in early AD vs. cognitively normal, age-matched controls. Second, and as also previously shown by PET, this global CMRg deficit in AD was primarily confined to the parietal cortex, posterior cingulate and thalamus. Third, neither ^11^C-AcAc uptake (CMRa) nor the AcAc uptake constant (*Ka*) were significantly different in the brain as a whole or in any brain region in AD or MCI vs. the age-matched cognitively healthy controls (**Figure 7**). Fourth, plasma AcAc and CMRa were significantly positively correlated, the slope of which did not differ between early AD and cognitively healthy age-matched controls or young adults (Castellano et al., 2015b; **Figure 5**). Since brain ketone utilization in AD was proportional to plasma concentration and this relationship had the same slope as in age-matched controls (**Figure 7**), we conclude that brain ketone uptake is not significantly disrupted early in AD.

As our database grows, we see a trend emerging toward a lower AcAc uptake constant for the brain *as a whole* in cognitively normal older people vs. young adults. However, when analyzed region by region, the results are not significant but pooled for the whole brain they are (**Figure 9**). So far, there is no trend toward lower brain ^11^C-AcAc uptake or a lower AcAc uptake constant in MCI or AD compared to our healthy older controls. Hence, caution is needed in comparing either brain ketone uptake results across age groups or in aging-associated cognitive decline for the whole brain vs. measurements focused more on major brain regions, specific nuclei or parts of the cortex.

## Complimentary Approaches to Increase Ketogenesis?

**FIGURE 10:**
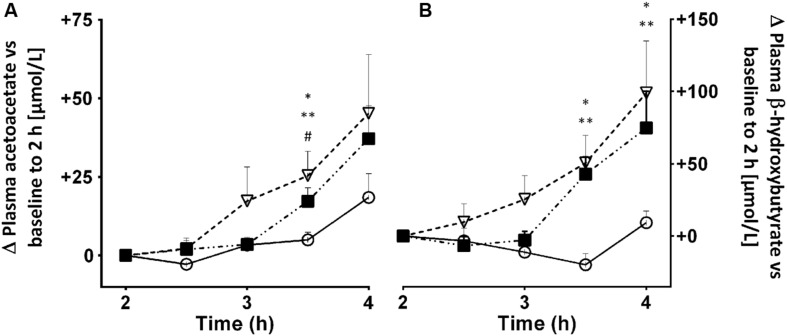
**Plasma acetoacetate **(A)** and β-HBA **(B)** 2–4 h after an oral dose of 2.5 (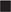) or 5.0 mg/kg (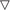) of caffeine vs. no treatment control (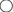).** Plasma caffeine reached a plateau 1–2 h after being consumed (data not shown), hence the reason for starting the ketone measurements at 2 h post-dose. Values are mean ± SEM (*n* = 10/point, with each participant undertaking each of the three treatments). Ketone data were normalized to zero at 2 h. ^∗^ Control vs. 2.5 mg/kg (^∗^*p* < 0.05) vs. 5.0 mg/kg (^∗∗^*p* < 0.05), and caffeine 2.5 vs. 5.0 mg/kg (^#^*p* < 0.05).

We have investigated whether substances that increase fatty acid availability to or oxidation by the liver might also increase ketogenesis. If so, this would have the potential benefit of either reducing the dose of MCT needed thereby reducing side-effects, or increasing the ketogenic effect of the same dose of MCT. Three such substances we have tested are alpha-linolenic acid (18:3n-3) and bezafibrate, two PPAR-alpha stimulators, and caffeine, which stimulates lipolysis thereby raising plasma free fatty acids (Acheson et al., 2004). Alpha-linolenic acid is also potentially ketogenic because it is the most beta-oxidized of the common dietary long chain fatty acids (Mamelak, 2012). Consuming 2 g of alpha-linolenic acid daily in the form of flaxseed over 4 weeks didn’t change overnight fasting plasma ketones. However, it did raise post-prandial ketone production by 26% but only in young adults; there was no significant effect in older adults (Hennebelle et al., 2016). Treatment with 400 mg of bezafibrate daily for 12 weeks was mildly ketogenic and increased fatty acid oxidation. Bezafibrate reduced plasma insulin and glucose suggesting that it may have a mild insulin-sensitizing effect. Plasma long chain fatty acids were also significantly lower after bezafibrate (Tremblay-Mercier et al., 2010). Co-treatment with bezafibrate (400 mg/d for 8 weeks) and 60 g/day of MCT transiently increased AcAc/β-HBA more than MCT alone (Courchesne-Loyer et al., 2015). A 2.5 or 5.0 mg/kg dose of caffeine taken with a small breakfast was moderately ketogenic 2–4 h post-dose. During the 4 h test period, plasma ketones and free fatty acids rose significantly more on caffeine than in the control test and the rise was broadly proportional to the dose of caffeine (**Figure 10**). We have not yet explored whether caffeine affects the ketogenic effect of MCT. Overall, these effects are modest but may merit further investigation in a clinical population.

## Neuronal Hyperexcitability, Epileptic Seizures and AD

Epileptic seizures are more prevalent in the aging population that at any other age, a situation that seems to be related to a higher incidence of cerebrovascular disease in older people. Sporadic or late-onset AD is associated with higher risk of seizures than in the general population (Amatniek et al., 2006; Vossel et al., 2013; Zilberter et al., 2013). Higher genetic or familial risk of AD is associated with hippocampal hyperexcitability and may be linked to neuronal loss and reorganization and to greater medial temporal lobe atrophy than in controls (Lapointe et al., 2016). Other factors that may contribute to this hyperexcitability include weakening of the inhibitory effect of GABA, and neuronal hypometabolism in the brain (Zilberter et al., 2013). Hyperexcitability in turn can further disrupt brain energy metabolism thereby contributing to a vicious cycle of deteriorating brain function and energy exhaustion. Given the efficacy of ketogenic treatments in controlling refractory epileptic seizures, especially in children, and their emerging utility for cognitive decline in AD, it would therefore seem to be worth assessing the efficacy of ketogenic treatments for seizures in the geriatric population.

## Issue – is Mitochondrial Function Impaired Early in AD?

The bulk of the ATP needed during energy metabolism is produced by oxidative phosphorylation in mitochondria. Glucose can also produce some ATP via glycolysis, a process occurring outside mitochondria, whereas ketones produce ATP uniquely via oxidative phosphorylation. Mitochondrial damage and increased production of reactive oxygen species have been proposed to underlie beta-amyloid accumulation and cognitive deterioration in AD (Gibson et al., 2000; Swerdlow and Khan, 2004; Yao et al., 2009; Swerdlow et al., 2014). Ketone catabolism is entirely mitochondrial, so normal whole body ketone oxidation in older humans (Freemantle et al., 2009) argues for largely intact mitochondrial function in reasonably healthy older people. Normal brain ketone metabolism in AD (**Figure 7**) also suggests that the enzymes of mitochondrial oxidative phosphorylation in the brain continue to function relatively normally, at least early in AD. Hence, early in AD, the problem with brain glucose metabolism is not necessarily at the mitochondrial level but possibly more because of lower glycolysis to acetyl CoA (see Issue – Brain Glucose Uptake or Glycolysis or Both?). Lower production of acetyl CoA would impair neuronal function thereby accounting for the well-known observation of lower glucose transport into the brain in AD. Oxidative damage to mitochondria and mitochondrial dysfunction would also tend to increase.

The ^11^C-AcAc used in our PET studies is chemically identical to AcAc produced by the body and is metabolized to ^11^C-CO_2_. Since the combined process of both brain uptake and metabolism of ^11^C-AcAc is still normal in early AD, and since ketone metabolism is uniquely mitochondrial, these results indirectly support the speculation nearly 30 years ago by Hoyer et al. (1988) that oxidative phosphorylation and therefore mitochondrial function is relatively normal early in AD. Such an interpretation is also supported by the clinical studies showing better cognitive scores in AD when more ketones are provided to the brain (**Table 6**) because otherwise ketones would not have clinical benefit.

The very high fat ketogenic diet increases various parameters that represent the overall energy status of the brain, including ATP and brain/blood glucose (DeVivo et al., 1978; Veech et al., 2001; Cahill, 2006), as well as citric acid cycle activity (Roy et al., 2015). Neural protection by ketones may also be related to improved mitochondrial biogenesis and improved respiratory function (Bough et al., 2006), as well as reduced mitochondrial production of reactive oxygen species in response to glutamate (Maalouf et al., 2009). Since oxidative phosphorylation in mitochondria generates free radicals and ketone metabolism is uniquely oxidative, it could also be argued that ketogenic supplements should actually make mitochondrial dysfunction *worse*, which should in turn cause cognitive deterioration. This doesn’t happen so, again, mitochondrial function can apparently cope with the increased oxidative load caused by metabolizing ketones. Nevertheless, this topic definitely needs further investigation.

## Issue – Brain Glucose Uptake or Glycolysis or Both?

The magnitude of the lower glucose metabolism by the AD brain was well-established by arterio-venous difference studies done 20–30 years ago (Lying-Tunell et al., 1981; Hoyer et al., 1988; Ogawa et al., 1996). However, these studies could not establish whether the glucose problem was with glucose transport into the brain via GLUT, or with glycolysis, i.e., the metabolism of glucose to pyruvate within the brain, or both. PET studies clearly show that glucose (FDG) uptake into the brain and its conversion to glucose-6-phosphate by hexokinase is lower in AD. However, FDG-PET cannot establish whether the glycolytic steps are also impaired. Since mitochondrial function seems to be normal in early AD and glucose transport in the brain is dependent on neuronal activity, the brain glucose hypometabolism problem in AD seems at least initially to be with glycolysis because several glycolytic enzymes are impaired in AD, including phosphofructokinase (Bowen et al., 1979; Iwangoff et al., 1980), alpha-ketoglutarate dehydrogenase complex (Gibson et al., 2000), and pyruvate dehydrogenase (Perry et al., 1980; Sorbi et al., 1983; Cunnane et al., 2011). If the problem in AD starts with deteriorating glycolysis, neural viability would eventually decrease, which would in turn decrease glucose transport into the brain because it depends on neural activity (Cunnane et al., 2011). As proposed many years ago by Hoyer et al. (1988), lower glycolysis to acetyl CoA would increase the brain’s dependence on other routes to generate ATP, including lactate and possibly even gluconeogenesis. The brain’s dependence on these routes to generate ATP could help account for the adverse changes in brain amino acid metabolism and neurotransmitter production including acetylcholine (Hoyer et al., 1988). Hence, our perspective is that the AD brain must be gradually pushed toward starvation mostly due to deteriorating glycolysis.

## Issue – Is Excessive Cataplerosis An Impediment to Successful Keto-Neurotherapeutics?

In addition to generating ATP, the citric acid cycle also has a key role in providing intermediates for several brain molecules including the neurotransmitters, gamma-aminobutyric acid and acetylcholine. The use of intermediates in the citric acid cycle to make molecules other than ATP is known as *cataplerosis* (Owen et al., 2002). Cataplerosis is usually balanced by *anaplerosis*, which is the net contribution of carbon from various sources to synthesize molecules derived from intermediates in the citric acid cycle. Glucose and oxaloacetate are anaplerotic so when both are insufficiently available, cataplerosis rapidly depletes the citric acid cycle (Wilkins et al., 2016). Unlike glucose, glutamine, pyruvate and precursors to propionyl CoA, the four carbon ketones (AcAc and β-HBA) do not contribute any carbon to anaplerosis (Brunengraber and Roe, 2006). Indeed, ketones are probably cataplerotic in part because they increase citric acid cycle activity (Roy et al., 2015). Hence, glucose itself or an alternative anaplerotic substrate is essential in order to metabolize ketones, especially as ketosis becomes more extreme.

When glucose supply to the brain is severely limited, such as in inherited GLUT-1 deficiency, there is insufficient glucose entering tissues to support energy production. Providing a ketogenic supplement is clinically beneficial but without anaplerotic input, chronic ketosis could potentially exhaust the citric acid cycle (Mochel et al., 2005; Brunengraber and Roe, 2006; Roe and Mochel, 2006). Triheptanoin (triglyceride with three heptanoic acids) is an odd-carbon MCT that is both ketogenic and anaplerotic and has clinically significant beneficial effects in GLUT-1 transporter deficiency and in Huntington’s disease (Mochel et al., 2005, 2010; Pascual et al., 2014). Furthermore, ketogenesis in the liver requires about 150 g/day of glucose that needs to be supplied by gluconeogenesis (Garber et al., 1974; Fukao et al., 2004). An alternative route of gluconeogenesis of unknown importance during nutritional ketosis involves increased acetone production which can be converted to glucose (Owen and Reichard, 1971; Reichard et al., 1974), thereby potentially contributing to sustaining both ketogenesis and anaplerosis.

This question of ketogenesis and anaplerosis is relevant to brain hypometabolism in AD because as AD becomes more severe, brain glucose uptake and/or utilization continue to deteriorate thereby further compromising both energy production and anaplerosis which are both needed for neurotransmitter synthesis. This adverse situation could potentially improve or worsen with sustained ketosis; it all depends on the trade-off between supplying more ketones to compensate for the glucose deficit and generate ATP, vs. burning out the citric acid cycle and depleting acetylcholine and GABA if cataplerosis exceed anaplerosis. Nevertheless, ketones are not the only fuel that bypasses impaired glycolysis: recent animal studies suggest that direct administration of pyruvate could be beneficial component of a ketogenic intervention for AD (Zilberter et al., 2013). *In vitro* studies show that exogenous oxaloacetate may also help bypass issues with glycolysis and maintain mitochondrial respiration (Wilkins et al., 2016).

## Issue – Is the Ketogenic Response to All MCT the Same?

Medium chain triglyceride are a generic product that varies widely in composition. They are usually concentrated in caprylic and capric acids but the ratio of these two fatty acids can vary from 70:30 to 30:70. Despite this variability, a compilation of various studies shows a significant positive correlation between the oral dose of MCT given and the resulting maximal plasma ketone response (**Figure 6**). Although MCT are most commonly a combination of caprylic and capric acids, these two fatty acids are also available separately. In unpublished work, we have observed that the net plasma ketone response to an equal dose of essentially pure caprylic acid exceeds by 15–20% that of an MCT containing caprylic:capric acid at 60:40 which in turn exceeded that of coconut oil by a wide margin (manuscript in preparation). At the same time, the change in plasma AcAc/β-HBA was actually significantly higher for coconut oil than for caprylic acid. This was an acute metabolic study conducted during an 8 h period with a 20 ml dose of the test ketogenic substance taken at breakfast and a second 20 ml dose taken at mid-day without a meal; whether the same changes occur in the long-term still needs to be evaluated.

**FIGURE 11:**
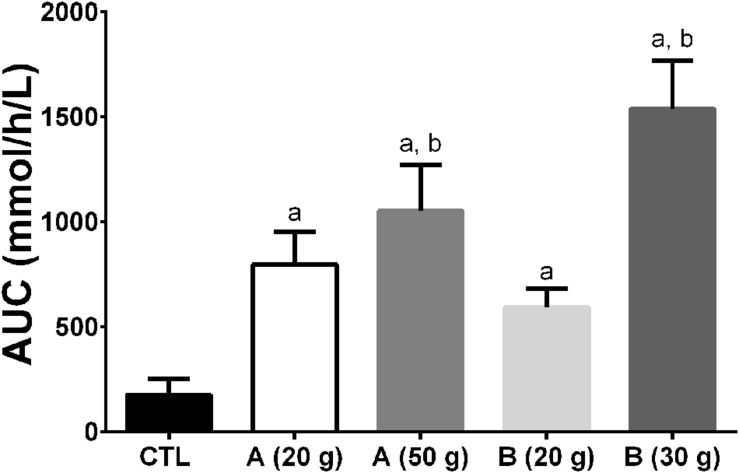
**Area under the curve (AUC) of plasma ketone response (beta-hydroxybutyrate + acetoacetate) after ingestion of two different MCT emulsions **(A,B)** during a 4 h metabolic study day.** All the treatments differed from the Control (a – *p* ≤ 0.05). The 30 g dose of product B and the 50 g dose of product A differed from the 20 g dose of product B (b – *p* ≤ 0.05). Interestingly, the 50 g dose of product A gave a 40% lower AUC than the 30 g dose of product B demonstrating that different homogenates of MCTs can induce widely differing plasma ketone responses, at least in an acute study design. Proprietary considerations prevent providing a full description of products A and B.

The formulation of the oral dose of MCT may also impact on the ketone response. We have observed that a 30 g dose of MCT in one formulation can generate a 40% higher plasma ketone response over 4 h than a different formulation containing 50 g of MCT (**Figure 11**). These results raise the questions of whether it matters which MCT is consumed, the bioequivalence of coconut oil vs. MCT, and what metric is most important in assessing the metabolic response to ketogenic supplements; β-HBA, AcAc, both combined, their ratio, or something else.

## Issue – Metabolic Phenotype of the Cognitively Healthy Older Person or Red Flag?

Age-normalization is a standard procedure with cognitive scores for older people and in MCI and AD. Hence, a lower raw cognitive score in an older person is not necessarily indicative of progression toward AD; rather, it depends on the type of test and the degree that the score is lower relative to age-normalized values. In the same vein, should regional brain glucose uptake (CMRg) also be normalized for age because CMRg too declines with age even in cognitively healthy older persons? In order to determine whether a lower CMRg value with age is normal or represents a risk for AD, age-normalized reference values need to be established so as to be able to distinguish a physiologically normal from a pathological change in CMRg.

Changes in peripheral glucose metabolism leading toward insulin resistance and type 2 diabetes increase the risk of AD (Craft, 2009, 2012; Baker et al., 2011). As with low cognitive scores, should metabolic parameters be considered in including or excluding older persons from a reference group of cognitively healthy older people? In other words, from an energy metabolism perspective, how should we define ‘normal’ or ‘healthy’ brain aging? The answer affects not only how data on brain energy metabolism in MCI and AD are interpreted but whether lower brain energy metabolism during ‘normal’ aging is truly physiological or imminently pathological. Such age-normalization would have two goals – to facilitate the early diagnosis of AD and, if such age-normalized brain glucose hypometabolism were present, to implement a pre-emptive intervention, whether ketone-based or other. One of the challenges with age-normalization of cognitive or metabolic data is that those in whom the onset of AD is imminent cannot presently be distinguished from those who may not get it for 10–20 years (or at all).

Our work shows that older people classified as cognitively normal by conventional neuropsychological tests corrected for age and education have significant brain atrophy, cortical thinning and lower brain glucose metabolism compared to cognitively normal younger adults (Nugent et al., 2014b, 2016). The differences were region-specific and more widespread for regional gray matter atrophy and cortical thinning than for CMRg. The age-related difference in CMRg was similar to the decline in volume and cortical thickness, and represents a decline of 0.3–0.4%/year (Nugent et al., 2016). Our work confirms several previous reports and suggests that the most consistent finding for CMRg during normal aging is glucose hypometabolism in the frontal cortex (De Santi et al., 1995; Moeller et al., 1996; Petit-Taboue et al., 1998; Garraux et al., 1999; Herholz et al., 2002; Zuendorf et al., 2003; Kalpouzos et al., 2009; Nugent et al., 2014a,b). Lower CMRg in the frontal cortex may be contributing to less efficient executive function in older people, i.e., greater recruitment or less inhibition of frontal regions for the same task than in younger adults (Hedden and Gabrieli, 2004). However, our older group still had cognitive test scores that were normal-for-age.

Quite a few neurometabolic differences can be expected between cognitively healthy older adults (**Table 3**). There were no diabetics or pre-diabetics in either group but our measure of insulin resistance, the HOMA2-IR, tended toward the high end of the normal range and was positively associated with higher CMRg, but only in the older group (Nugent et al., 2016). A HOMA2-IR toward the high end of the normal range and body fat content of at least 30% were both associated with normal cognitive function in older adults in this particular population (Nugent et al., 2016). Do these metabolic differences with age suggest that higher plasma insulin and a higher metabolic rheostat are necessary to maintain normal cognition in older people? Alternatively, do declining (though normal-for-age) cognitive scores with age represent a red flag because they drive up the metabolic rheostat? Either way, more work needs to be done evaluating the relation between changing metabolic-endocrine status and cognition regardless of age.

## Perspective

We make the case here that regional brain glucose hypometabolism can definitely be present in those at risk of AD but decades before the onset of cognitive decline associated with AD, i.e., that it is a pre-symptomatic problem (**Figure 3**). Hence, it is incorrect to perceive of brain glucose hypometabolism in AD as being uniquely a consequence of irreversible neuronal failure or death. Pre-symptomatic brain glucose hypometabolism isn’t necessarily the cause of AD or even the first step in the pathogenesis of AD. However, two points are clear – (i) AD is at least in part exacerbated by (if not actually caused by) chronic, progressive brain fuel starvation due specifically to brain glucose deficit, and (ii) attempting to treat the cognitive deficit early in AD using ketogenic interventions in clinical trials is safe, ethical, and scientifically well-founded (Henderson et al., 2009; Rebello et al., 2015; see also ClinicalTrials.gov).

A number of issues have been flagged here that will require further work in order to optimize keto-neurotherapeutics in AD. They include the state of brain mitochondrial respiration, whether the problem with brain glucose utilization starts with impaired glycolysis, the importance of balancing anaplerosis and cataplerosis, differential ketone responses to MCT mixtures depending on formulation, and the significance of lower CMRg in the frontal cortex during cognitively normal aging. The elephant in the room that we have intentionally not discussed till now is the beta-amyloid hypothesis of AD. Suffice it to note here that beta-amyloid accumulation occurs as a result of either impaired brain glucose availability to the brain (Velliquette et al., 2005) or impaired glycolysis within the brain (Meier-Ruge et al., 1994). Beta-amyloid also contributes to impaired glycolysis in the AD brain (Meier-Ruge and Bertoni-Freddari, 1997). Ketogenic treatments reduce amyloid burden in animal models of AD (Kashiwaya et al., 2000; Hertz et al., 2015; Yin et al., 2016) but this extremely encouraging effect remains to be verified in humans. Clinical trials with ketogenic interventions in AD, MCI or insulin-induced hypoglycemia start to improve some cognitive outcomes within hours to days (Hasselbalch et al., 1996; Reger et al., 2004; Henderson, 2008; Newport et al., 2015), arguing that if they also reduce amyloid burden, it is a secondary effect. Hence, we feel that dealing with the brain energy deficit would help the brain metabolize beta-amyloid as it normally should.

The challenge that immune function and neuroinflammation may pose with respect to providing the brain with sufficient energy during aging also deserves comment. We and others have previously proposed that immunosenescence in older people can tip toward a pro-inflammatory condition. Since the activated immune system consumes a lot of energy, heightened immune surveillance during aging constitutes a potentially important energy sink in the body which can compete with the brain for a fixed (or declining) energy intake in older people. This situation is probably exacerbated by infection, inflammation, or other chronic response of the immune system. Under such circumstances, it takes little to tip the body’s energy intake more toward supporting the immune response and away from the brain, which then contributes to brain energy starvation and increased risk of cognitive decline during aging (Fulop et al., 2013, 2015). Apart from the greatly increased need for energy by activated immune cells, pro-inflammatory cytokines impair ketone production (Pailla et al., 2001), making the brain’s energy supply even more vulnerable. Hence, brain energy deficit, neuropathology, neuroinflammation and cognitive decline all feed off each other in a vicious cycle that can lead to rapid progression of AD (**Figure 3**). Ketogenic treatments help elicit a protective response to neuroinflammation (Rahman et al., 2014), an encouraging observation that needs confirmation and further work.

Several different ketogenic interventions including prolonged fasting, a very high fat ketogenic diet containing no MCT, or a regular diet to which MCT or ketone esters all have a broadly similar neurological/cognitive benefit (**Table 6**). The cognitive benefit of ketones is observed under different conditions including in the presence of chronically impaired brain glucose availability or utilization due to disease (i.e., MCI or AD), or when the brain glucose deficit is acute, i.e., severe hypoglycemia experimentally induced by insulin infusion and starvation (Owen et al., 1967). The wide range of ketogenic conditions that protect cognition suggests that ketones themselves (rather than MCFAs) are central to their beneficial effect but this needs to be confirmed along with determining the actual mechanism. At present, a keto-neurotherapeutic approach is a safe and scientifically well-supported strategy to bypass deteriorating brain energy metabolism arising during aging; time will tell whether it is truly clinically effective to limit the onset or progression of cognitive decline in AD.

## Author Contributions

SC, AC-L, C-AC participated in the conception and drafting of the work. All authors participated in the revising and final approval of the content of this review.

## Conflict of Interest Statement

Some MCT used in our more recent studies was provided by Abitec Corporation. SC has participated in *ad hoc* consulting for Keto Products and Bulletproof. All the other authors declare that the research was conducted in the absence of any commercial or financial relationships that could be construed as a potential conflict of interest. The reviewer DR and handling Editor declared their shared affiliation, and the handling Editor states that the process nevertheless met the standards of a fair and objective review.

## Abbreviations

AcAc: acetoacetate
AD: Alzheimer’s disease
CMRa: cerebral metabolic rate of acetoacetate
CMRg: cerebral metabolic rate of glucose
FDG: ^18^F-fluorodeoxyglucose
GLUT: glucose transporter
β-HBA: beta-hydroxybutyrate
HOMA2-IR: homeostatic model of insulin resistance (version 2)
MCFA: medium chain fatty acid
MCT: medium chain triglyceride
PCOS: polycystic ovary syndrome
PET: positron emission tomography
